# MFA-CNN: An Emotion Recognition Network Integrating 1D–2D Convolutional Neural Network and Cross-Modal Causal Features

**DOI:** 10.3390/brainsci15111165

**Published:** 2025-10-29

**Authors:** Jing Zhang, Anhong Wang, Suyue Li, Debiao Zhang, Xin Li

**Affiliations:** 1 School of Electronic Information Engineering, Taiyuan University of Science and Technology, Taiyuan 030024, China; ahwang@tyust.edu.cn (A.W.); lsy@tyust.edu.cn (S.L.); 2021044@tyust.edu.cn (D.Z.); 2 Microelectronic Equipment Department, The 2nd Research Institute of China Electronics Technology Group Corporation, Taiyuan 030024, China; 15271904775@163.com

**Keywords:** EEG, fNIRS, emotion recognition, Granger causality, modality–frequency attention mechanism

## Abstract

**Background/Objectives:** It has become a major direction of research in affective computing to explore the brain-information-processing mechanisms based on physiological signals such as electroencephalography (EEG) and functional near-infrared spectroscopy (fNIRS). However, existing research has mostly focused on feature- and decision-level fusion, with little investigation into the causal relationship between these two modalities. **Methods:** In this paper, we propose a novel emotion recognition framework for the simultaneous acquisition of EEG and fNIRS signals. This framework integrates the Granger causality (GC) method and a modality–frequency attention mechanism within a convolutional neural network backbone (MFA-CNN). First, we employed GC to quantify the causal relationships between the EEG and fNIRS signals. This revealed emotional-processing mechanisms from the perspectives of neuro-electrical activity and hemodynamic interactions. Then, we designed a 1D2D-CNN framework that fuses temporal and spatial representations and introduced the MFA module to dynamically allocate weights across modalities and frequency bands. **Results:** Experimental results demonstrated that the proposed method outperforms strong baselines under both single-modal and multi-modal conditions, showing the effectiveness of causal features in emotion recognition. **Conclusions:** These findings indicate that combining GC-based cross-modal causal features with modality–frequency attention improves EEG–fNIRS-based emotion recognition and provides a more physiologically interpretable view of emotion-related brain activity.

## 1. Introduction

As global brain science research progresses, exploring the information-processing and cognitive mechanisms of the human brain through physiological signals in affective computing [1,2,3,4] has become increasingly prominent. Generally, physiological signals associated with neural activity fall into two categories [5,6]: one is the electrophysiological signals, such as electroencephalography (EEG), magnetoencephalography (MEG); and the other is signals of metabolic changes, such as functional near-infrared spectroscopy (fNIRS) and functional magnetic resonance imaging (fMRI). In the field of emotion recognition, EEG and fNIRS are often favored over MEG and fMRI, owing to their portability, cost-effectiveness, and ease of operation [7,8]. In particular, the simultaneous acquisition of EEG–fNIRS signals, which provides more comprehensive brain information than does single-modality data [9,10], has become a popular topic in emotion recognition research.

Research on neurovascular coupling (NVC) mechanisms posit [11,12] that neural activity in the brain is accompanied by the electrical activity and corresponding hemodynamic changes. In response to external stimuli, neuronal activation triggers electrical activity while simultaneously increasing local cerebral blood flow to meet the increased oxygen demands. This leads to an elevation in the intravascular concentration of oxyhemoglobin (HBO_2_) and a reduction in deoxyhemoglobin (HbR) [13,14]. Therefore, EEG reflects neural electrical activity with high temporal resolution, while fNIRS reveals metabolic processes through changes in blood oxygenation, offering superior spatial localization capabilities compared to EEG. Together, EEG and fNIRS provide a complementary toolset for investigating the neural mechanisms of emotion-related brain activity because of their temporal and spatial complementarity.

However, the temporal and spatial differences between EEG and fNIRS also present challenges for joint analysis [15,16,17,18]. Existing research has predominantly addressed this challenge by feature-level or decision-level fusion [19,20,21,22], which makes it difficult to reveal the potential coupling mechanisms between the two signals. EEG and fNIRS respectively reflect neural electrical activity and blood oxygenation dynamics in response to the same stimulus, suggesting a potential causal relationship between them. Therefore, it is necessary to employ measures that can quantitatively characterize causal direction and strength. Granger causality (GC), originally proposed in economics [23,24], has been widely used in neuroscience to explore the causal connections of physiological signals such as EEG, fNIRS, and fMRI [25,26,27,28]. There remains a relative paucity of systematic studies on cross-modal causality between EEG and fNIRS. Thus, this paper applies the GC method to analyze in depth the causal coupling between EEG and fNIRS signals during emotion induction, which is crucial for revealing mechanisms of emotional processing in the human brain and optimizing emotion recognition performance.

Furthermore, the EEG features, fNIRS features, and the combinations of different frequency bands of EEG and fNIRS features contribute differently to affective representations. Traditional methods often consider all modalities and frequency bands equally, ignoring differences in informativeness and limiting overall performance. In recent years, attention mechanisms have demonstrated advantages in multimodal fusion due to their ability to dynamically allocate weights based on feature importance. Inspired by this, we introduce a modality–frequency attention (MFA) mechanism based on cross-modal causal features to adaptively highlight modality and frequency-band features that are more sensitive to emotional states, thereby further enhancing recognition accuracy and robustness.

Above all, although the EEG or fNIRS alone can provide insights into human emotion brain activity, the complexity of brain function means that single-modality approaches are inherently limited. Therefore, this paper focuses on synchronously acquired EEG–fNIRS signals, and the primary contributions include the following:

- We proposed a novel causal metric for EEG-fNIRS to quantify their causal relationship.
- We designed an MFA-CNN emotion recognition model that integrates a 1D–2D CNN framework with a modality–frequency attention mechanism, which assigns weights to EEG, fNIRS, and cross-modal features to enhance recognition performance.
- We conducted systematic experiments on the TYUST-3.0 EEG-fNIRS multimodal emotion dataset [29] to evaluate MFA-CNN, including unimodal and multimodal settings, ablation studies, and comparisons with alternative classifiers.

The rest of the paper is organized as follows. Section 2 discusses the related work. Section 2 discusses the related work. Section 3 introduces EEG-fNIRS data acquisition and preprocessing. Section 4 details the proposed MFA-CNN model. Section 5 presents the experimental setup, results, and analysis. Section 6 concludes the paper and outlines future research directions.

## 2. Related Works

In this section, we introduce the related works involved in this paper, including the GC method, EEG electrode arrangement strategy, and multimodal emotion recognition network.

### 2.1. The GC Method

The basic idea of the GC method is that if the prediction error of the future moments of a time series X can be reduced by introducing the historical information of another time series Y, then the time series Y is assumed to have a causal relationship with the time series X. In recent years, researchers have explored the causal relationships between EEG, fNIRS, and combined EEG-fNIRS signals in numerous fields including workload, emotion recognition, motor control, cognitive assessment, and rehabilitation [30,31,32,33,34,35]. In EEG research, Guo et al. [36] used the sparse group lasso-Granger to construct an EEG causal brain network, which was then used to analyze the changes in human affective states. Bagherzadeh et al. [37] utilized a frequency domain GC method to construct EEG brain networks and used them to study the connectivity and differences of the brain under different emotions. Zachary et al. [38] applied GC analysis to determine how an emotion is affected by a person’s cultural background and situation. In the fNIRS research, Hu et al. [39] combined conditional GC analysis and fNIRS neuroimaging technique to examine the differences in brain activation and effective connectivity between calculation, planning, and reasoning and discovered that the performance of planning and reasoning was correlated with the activation in frontal cortex and parietal cortex, respectively. Lee et al. [40] investigated cerebral cortex activation during active movement and passive movement by using fNIRS signals.

In EEG-fNIRS research, Al-Shargie et al. proposed an ROC-based decision fusion of α-frequency EEG and fNIRS signal for improving the detection rate of mental stress [30]. Nour et al. designed a multiple bandwidth method with optimized CNN model to fusion the time–frequency features of EEG and fNIRS signals and to enhance the recognition performance of motor imagery [31]. Saadati et al. down-sampled the original EEG signals to match the sampling rate of the fNIRS signals and then input the simultaneous EEG-fNIRS signals into a CNN network, which increased the recognition performance of the workload memory tasks by 20% and 7% compared with the single-modal EEG and fNIRS signals, respectively [32]. Sun et al. adopt the feature-stitching method to integrate the time–frequency features of the different frequency EEG signals and fNIRS signals, whose emotion recognition performance was improved by 8% to 18% compared with single-modal signals [33]. Liang et al. adopted coherence and Granger causality (GC) between the EEG and fNIRS signals as the degree of neurovascular coupling to explore the causality relationship between neurophysiology and hemodynamics in children and adults during general anesthesia [34]. In [35], Kamat et al. constructed the GC brain network of EEG-fNIRS signals, to explore the differences in the flow of information between the experts and novices during the performance of the fundamentals of laparoscopic surgery.

### 2.2. Multimodal Emotion Recognition Network

With the advancement of deep learning, various attention mechanisms have been gradually integrated into recognition models such as CNN and CRNN to further explore the time–frequency characteristics in EEG and fNIRS signals related to emotion. For example, Hamidi et al. [41] combined Transformers with graph convolutional networks (GCNs) to capture the spatio-temporal features of EEG signals. In [42], a multi-channel hybrid fusion network based on EEG-fNIRS signals was proposed, which employs an efficient attention mechanism module to integrate the spatial information of EEG and fNIRS signals. In [43], an early fusion strategy based on deep learning was proposed to combine the spatial weight coefficients obtained from the fNIRS with EEG features and to adaptively allocate the weights coefficients according to their learned importance.

Moreover, the attention mechanism has also been introduced in the frequency domain of EEG to highlight the differential contributions of frequency bands to emotional states. The frequency–spatial attention mechanism proposed in [44] simultaneously models weight distributions across both frequency and spatial domains. In [45], a multi-band graph feature fusion method was proposed to enhance classification performance by integrating cross-band information. In summary, attention-enhanced multimodal networks have demonstrated clear benefits for EEG-fNIRS emotion recognition, providing a solid basis for integrating GC-derived cross-modal causal features into end-to-end models.

## 3. Materials

### 3.1. Participants

Fifty healthy, right-handed undergraduate students (25 males and 25 females; age 22–26 years; mean 24.1 years) participated in the experiments. None had a history of mental or other brain-related illnesses. Before each experiment, the participants were briefed about the purposes and procedures of the experiments. All participants provided informed consent and received financial compensation for their involvement. The experimental protocol adhered to the Declaration of Helsinki for human experimentation and was approved by the Taiyuan University of Technology Research Ethics Committee.

### 3.2. Experimental Design

**Figure 1:**
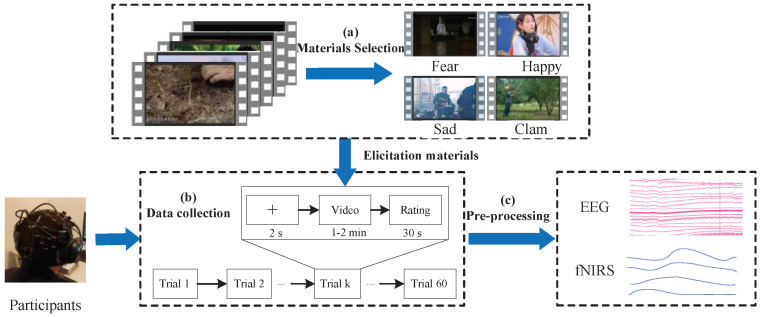
The framework of EEG-fNIRS signal acquisition.

Figure 1 overviews the EEG-fNIRS acquisition pipeline for emotion induction, including stimulus selection, synchronized signal recording, and preprocessing.

#### 3.2.1. Stimulus Selection

Compared with static images or speech, video clips elicit stronger affective responses. We first curated 120 video clips with diverse emotions from films and documentaries, and each clip lasted approximately 1–2 min. Independent volunteers then assessed each clip on a 5-point Likert scale for valence (pleasure), arousal, and dominance based on their felt experience. Based on these ratings, we compiled a final stimulus set comprising four emotion categories, with each category including 15 video clips: happy, fear, sad, and calm.

#### 3.2.2. Acquisition Equipment

**Figure 2:**
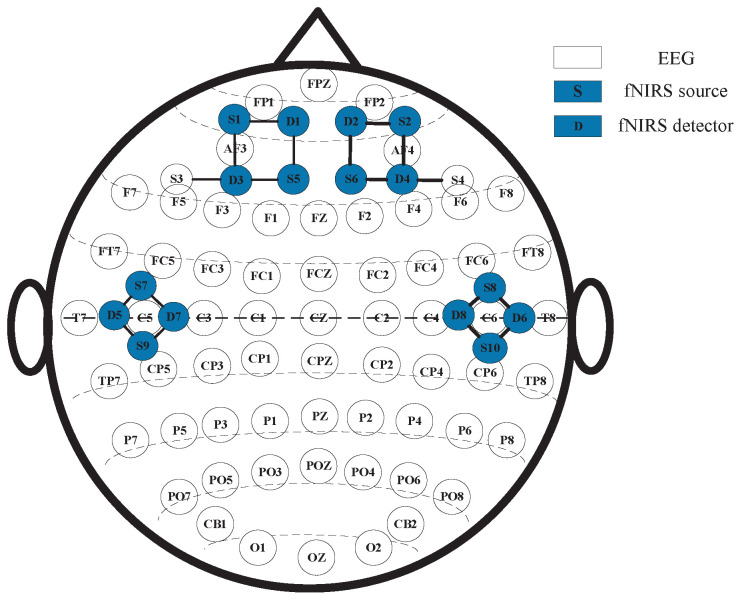
The arrangement and layout of EEG electrodes and fNIRS optodes.

As for the EEG-fNIRS acquisition, the EEG electrodes and fNIRS optodes were placed at non-overlapping scalp locations. EEG was recorded using Neuroscan SynAmps2 (Neuroscan, Neuroscan USA, Ltd., Charlotte, NC, USA), with 62 electrodes positioned according to the international 10–20 system, and sampled at 1000 Hz, covering the whole scalp. fNIRS was recorded using NirSmart (DanYang HuiChuang Medical Equipment Co., Ltd., Danyang, China), with 10 sources and 8 detectors, operating at 760/850 nm and 11 Hz. Given the key role of the frontal and temporal cortices in emotion processing, we placed 6 sources/4 detectors over the temporal region and 4 sources/4 detectors over the frontal region. The details of the EEG electrodes and fNIRS optodes are illustrated in Figure 2, where S denotes a source, D denotes a detector, and each S–D denotes pairing a measurement channel.

#### 3.2.3. Data Collection

To ensure high data quality, experiments were conducted in a shielded laboratory to minimize electromagnetic and environmental noise. During the experiment, participants sat comfortably and received both visual and auditory stimuli: videos were presented on an LCD monitor at about a 1 m viewing distance, and audio was delivered via high-fidelity, head-mounted headphones. The protocol was programmed in E-prime 3, and each participant completed 60 trials.

The experimental procedure is shown in Figure 1b. It commenced with a “+” symbol displayed on the screen for 2 s to prompt the participant to focus on the upcoming emotional clip. Next, a selected emotional clip was randomly presented, and EEG and fNIRS signals were recorded synchronously. After each video clip, participants were given 30 s to assess their felt emotions in terms of pleasure, arousal, dominance, and emotion type. This step was essential for verifying whether the video clips effectively elicited the intended emotions.

### 3.3. Signal Pre-Processing

EEG preprocessing: EEG data were re-referenced, downsampled to 200 Hz, band-pass filtered (4–45 Hz), and baseline-corrected. Artifacts (e.g., ocular movements) were removed using independent component analysis (ICA). Time–frequency representations were obtained via the short-time Fourier transform (STFT), and band-specific features were extracted for θ (4–8 Hz),α (8–12 Hz),β (12–30 Hz), and γ (30–45 Hz).

fNIRS preprocessing: Major noise sources include cardiac pulsation (1–1.5 Hz) and respiration (0.2–0.4 Hz), whereas task-related hemodynamic components mainly lie within 0.005–0.21 Hz. Preprocessing comprised baseline correction, motion-artifact attenuation, and 0.01–0.2 Hz band-pass filtering. Changes in oxyhemoglobin (HbO_2_) and deoxyhemoglobin (HbR) concentrations (HbO_2_ and HbR) were computed using the modified Beer–Lambert law. To temporally align with EEG, the HbO_2_/HbR time series were upsampled to 200 Hz via envelope-based interpolation.

## 4. The Proposed MFA-CNN Method

**Figure 3:**
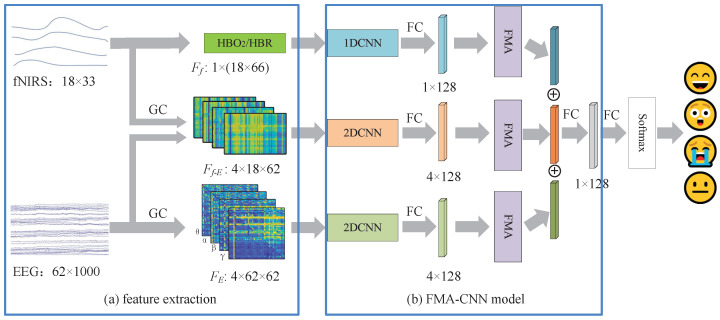
The framework of the proposed MFA-CNN method. where the blue blocks indicate the fNIRS feature processing branch, the orange blocks indicate the EEG–fNIRS cross-modal GC feature processing branch, the green blocks indicate the GC-based EEG feature processing branch, and the purple block denotes the MFA module.

Figure 3 illustrates the proposed MFA-CNN framework for emotion recognition. After preprocessing, three feature streams are extracted: (i) GC-based EEG connectivity matrices, (ii) fNIRS hemodynamic features (HbO_2_/HbR), and (iii) EEG–fNIRS cross-modal GC features. Each stream is fed into a modality-specific encoder—a 1D–CNN for the fNIRS time series and 2D-CNNs for the matrix-form inputs—to obtain deep representations. A modality–frequency attention (MFA) module then adaptively reweights and fuses the three streams, and a Softmax classifier outputs the final emotion results.

### 4.1. The GC Features of EEG Signal

According to Granger causality, for two time series $X_{t}$ and $Y_{t}$, if the prediction of $Y_{t}$ based on the joint past $\left\{X_{t-i},Y_{t-i}\right\}$ is better than that based only on $\left\{Y_{t-i}\right\}$, then *X* is said to Granger-cause *Y*. To illustrate, let $S_{X}\left(t\right)$ and $S_{Y}\left(t\right)$ denote two EEG time series. The univariate AR(*p*) model for $S_{X}\left(t\right)$ is

(1) $$S_{X}\left(t\right)=\sum_{i=1}^{p}\alpha_{i}S_{X}\left(t-i\right)+\mu_{1}\left(t\right)\;$$

The bivariate AR/VAR(*p*) model for ${\left[S_{X}\left(t\right),\;S_{Y}\left(t\right)\right]}^{⊤}$ is

(2) $$S_{X}\left(t\right)=\sum_{i=1}^{p}\alpha_{1i}S_{X}\left(t-i\right)+\sum_{i=1}^{p}\alpha_{2i}S_{Y}\left(t-i\right)+\mu_{2}\left(t\right)\;$$

where *p* is the model order, $a_{i},\;\alpha_{1i},\;\alpha_{2i}$ are autoregressive coefficients, and $\mu_{1}\left(t\right),\;\mu_{2}\left(t\right)$ denotes the prediction error of the model, respectively.

Let $V\mathrm{ar}(\mu_{1})$ and $V\mathrm{ar}(\mu_{2})$ denote the variances of the residuals in the restricted and full models, respectively. Then the GC effect from $S_{Y}\left(t\right)$ to $S_{X}\left(t\right)$ is defined as

(3) $$F_{Y\to X}=\ln\frac{V\mathrm{ar}(\mu_{1})}{V\mathrm{ar}(\mu_{2})}\;$$

If $F_{Y\to X}>0$, there exists a GC relationship between $S_{X}\left(t\right)$ and $S_{Y}\left(t\right)$, i.e., $S_{Y}\left(t\right)$ Granger-causes $S_{X}\left(t\right)$. The converse measure $F_{X\to Y}$ is defined analogously by interchanging the roles of $S_{X}\left(t\right)$ and $S_{Y}\left(t\right)$.

In summary, causal features of EEG in the θ, α, β, and γ bands can be computed via Equations (1)–(3). Similarly, the causal features of fNIRS signals can be computed.

### 4.2. The Causal Feature of EEG-fNIRS

In the previous section, the fNIRS signals were upsampled to match the EEG sampling rate, ensuring temporal alignment between the two modalities. Following the GC method, let $E_{Z}\left(t\right)$ denote the EEG time series at channel *z* and let $E_{f}\left(t\right)$ denote the fNIRS blood-oxygenation time series (HbO_2_ or HbR) at optode f. To test whether fNIRS Granger causes EEG, we compare a restricted AR model for $E_{z}\left(t\right)$ with a full bivariate AR model that additionally includes past fNIRS terms:

(4) $$E_{Z}\left(t\right)=\sum_{i=1}^{q}\beta_{i}E_{Z}\left(t-i\right)+\varepsilon_{1}\left(t\right)\;$$

(5) $$E_{Z}\left(t\right)=\sum_{i=1}^{q}\beta_{1i}E_{Z}\left(t-i\right)+\sum_{i=1}^{q}\beta_{2i}E_{f}\left(t-i\right)+\varepsilon_{2}\left(t\right)\;$$

where *q* denotes the model order (e.g., selected via AIC/BIC), $\beta_{i}$, $\beta_{1i}$ and $\beta_{2i}$ are the autoregressive coefficients, and $\varepsilon_{1}\left(t\right)$ and $\varepsilon_{2}\left(t\right)$ are the prediction errors of the restricted model in (4) and the full model in (5), respectively.

Accordingly, the GC from fNIRS to EEG is defined as the log ratio of the residual variances:

(6) $$F_{f\to E}=\ln\frac{V\mathrm{ar}(\varepsilon_{1})}{V\mathrm{ar}(\varepsilon_{2})}\;$$

**Figure 4:**
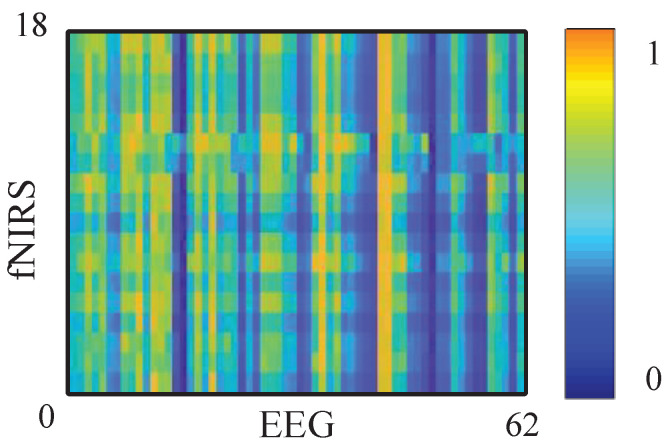
The GC matrix of fNIRS to EEG signal. Darker colors indicate lower GC values (weaker directed causal influence between EEG and fNIRS), whereas brighter colors indicate higher GC values (stronger causal coupling between the two modalities).

A larger $F_{f\to E}$ indicates that including past fNIRS improves the prediction of EEG; i.e., fNIRS Granger causes EEG at the tested pair (f,z). By repeating (4)–(6) for all fNIRS-EEG channel pairs, we obtain a channel-wise GC matrix $G_{f\to E}$. The reverse matrix $G_{E\to f}$ is computed analogously by swapping the roles of $E_{Z}\left(t\right)$ and $E_{f}\left(t\right)$, as shown in Figure 4.

### 4.3. The MFA-CNN Module

As described in Figure 3, the proposed MFA-CNN fuses the three feature streams, and each stream is first passed through a feature-specific CNN encoder composed of three convolutional layers (with 32/64/128 filters) followed by a fully connected (FC) layer with 128 units, outputting a 128-dimensional deep representation per stream. Pooling layers are intentionally omitted between adjacent convolutions to preserve the spatial structure that could be lost through repeated down-sampling. The three 128-dimensional vectors are then concatenated into a 384-dimensional fused vector, which is fed to a final FC layer and a Softmax classifier to produce the emotion label. Table 1 reports the detailed hyper-parameters of the MFA-CNN.

**Figure 5:**
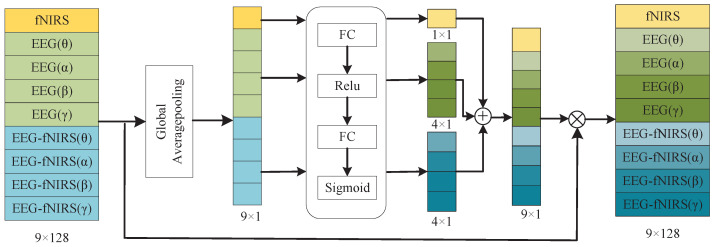
The framework of MFA module.

Prior research [30] has demonstrated different abilities in the emotion recognition of EEG signals across different frequency bands. Likewise, the GC-based cross-modal features between EEG bands and fNIRS exhibit varying discriminative power. To exploit this heterogeneity, we developed a modality–frequency attention (MFA) module that learns weights over modality–frequency combinations and rescales the corresponding deep features before fusion, as illustrated in Figure 5. In this way, features most informative of emotional state receive higher importance, improving both accuracy and robustness.

In the MFA module, we first apply global average pooling (GAP) along the feature dimension of the input $X\in^{\;C*D}$ (where C denotes modality–frequency channels, and *D* denotes features per channel). The GAP process results in a channel descriptor $s\in^{\;C*1}$:

(7) $$S=GAP\left(X\right)=\frac{1}{D}\sum_{d=1}^{D}D_{C,d}\;$$

Next, the descriptor vector $s\in R^{9\times 1}$ is partitioned into three groups: the EEG descriptors $s_{\mathrm{EEG}}\in R^{4\times 1}$, the fNIRS descriptor, $s_{\mathrm{fNIRS}}\in R^{1\times 1}$, and the EEG-fNIRS cross-modal descriptors, $s_{\mathrm{EEG}-\mathrm{fNIRS}}\in R^{4\times 1}$. For each group, attention weights are produced by a two-layer fully connected network with *ReLU* followed by *sigmoid*. The computations are given in Equations (8)–(10):

(8) $$z_{fNIRS}=\delta \left(W_{f2}\delta \left(W_{f1}s_{fNIRS}\right)\right)\;$$

(9) $$z_{EEG}=\delta \left(W_{e2}\delta \left(W_{e1}s_{EEG}\right)\right)\;$$

(10) $$z_{EEG-fNIRS}=\delta \left(W_{c2}\delta \left(W_{c1}s_{EEG-fNIRS}\right)\right)\;$$

where $W_{\cdot 1}$ and $W_{\cdot 2}$ are the weights of the two FC layers, and δ(·) and σ(·) denote the *ReLU* and *sigmoid* activations, respectively:

(11) δ(x)=max(x,0)

(12) $$\sigma \left(x\right)=\frac{1}{1+e^{-x}}\;$$

The resulting weights are broadcast back to the corresponding rows of *X* and applied by element-wise multiplication, and producing reweighted features ${\tilde{X}}_{\mathrm{EEG}}$, ${\tilde{X}}_{\mathrm{fNIRS}}$, and ${\tilde{X}}_{E↔F}$, which are then concatenated and forwarded to the classifier.

### 4.4. The Proposed Weighted Multi-Loss Function (WML)

To better exploit informative features for emotion recognition, we adopt a weighted multi-loss scheme to jointly optimize the MFA-CNN. We define the global loss of the fused prediction as $L_{\mathrm{global}}$ and the local loss from each modal-frequency combination as $L_{\mathrm{local}}$. The total loss is described as follows:

(13) $$L=L_{global}+\mu L_{local}\;$$

where μ balances the contributions of global and local supervision.

For the global term $L_{\mathrm{global}}$, we use cross-entropy between the ground-truth one-hot label $y\in {\left\{0,1\right\}}^{C}$ and the fused Softmax prediction $\hat{y}\in {\left[0,1\right]}^{C}$:

(14) $$L_{global}=H\left(y_{i},{\hat{y}}_{i}\right)=-\sum_{i=1}^{C}y_{i}\log{\hat{y}}_{i}\;$$

where *C* denotes the number of emotion categories.

For the local loss $L_{\mathrm{local}}$, we aggregate three branch-specific cross-entropies:

(15) $$L_{local}=w_{f}\times L_{fNIRS}+w_{e}\times L_{EEG}+w_{ef}\times L_{EEG-fNIRS}\;$$

where $L_{\mathrm{fNIRS}},\;L_{\mathrm{EEG}},\;L_{\mathrm{EEG}-\mathrm{fNIRS}}$ are computed from the corresponding branch outputs, and $w_{f},\;w_{e},\;w_{ef}\;\geq \;0$ are the weights reflecting the recognition strength of each branch.

In practice, the weights are updated from the branch accuracies $\left\{a_{f},\;a_{e},\;a_{ef}\right\}$ (e.g., running averages over training batches) and normalized to sum to one:

(16) $$w_{k}=\frac{a_{k}}{a_{e}+a_{f}+a_{ef}}\left(k=e,\;f,\;ef\right)\;$$

This weighting prioritizes branches with higher discriminative power while still allowing gradients from all branches to contribute to learning.

## 5. Experimental Results and Analysis

### 5.1. Parameter Settings

In this paper, all experiments were conducted under a subject–dependent setting. Specifically, our experiments were conducted on Windows 10 using Python 3.9, and PyTorch 2.0, running on an Intel Core i9-13900KF (3.0 GHz) CPU with an NVIDIA GeForce RTX 4090 GPU. For deep learning optimizer, the Adam optimizer is employed with a learning rate of 0.005 and batch size of 32, and a dropout rate of 0.3 is applied to mitigate overfitting.

For the original EEG and fNIRS signal, the raw streams were segmented with a 3 s sliding window and a 1.5 s overlap. In the subject-dependent setting, splits were performed clip-wise per participant to avoid leakage: for each emotion class, 12 clips were used for training and the remaining 3 clips were reserved for validation/test (applied before windowing so that no trial contributes windows to multiple splits). To evaluate the emotion recognition, 5-fold cross-validation is implemented across all 50 participants, and the resulting average recognition accuracy and standard deviation are used as evaluation metrics.

### 5.2. The Emotion Recognition Performance of Single Modal Features

**Figure 6:**
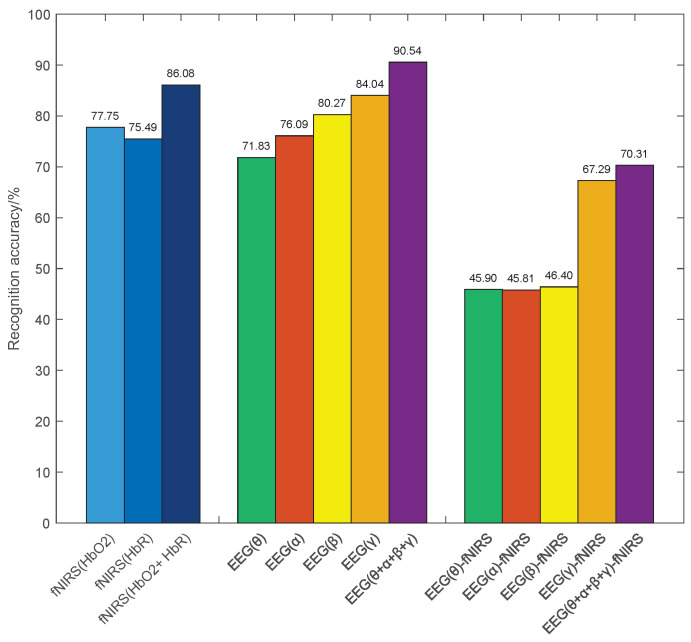
The emotion recognition rate of single features.

In this section, we describe the evaluation of single features extracted from EEG and fNIRS using the same CNN classifier, and the results are summarized in Figure 6.

(1) For fNIRS features, the average accuracies of HbO_2_ and HbR features are 77.45% and 75.49%, respectively. The cascaded HbO_2_ + HbR feature lifts performance to 86.08%, i.e., achieving an improvement of 8.33% and 10.59% over the single HbO_2_ and HbR features, respectively. These results indicate that the two hemoglobin components provide complementary information for emotion recognition.

(2) For the GC features of EEG signal, the average emotion recognition accuracies for the θ, α, β, and γ frequency bands are 71.83%, 76.09%, 80.27%, and 84.04%, respectively. Furthermore, the combined features achieve significant improvements over single-band features. This improvement can be attributed to the fact that EEG signals in different frequency bands express distinct emotional information, utilizing the complementarity between them enhances emotion recognition performance.

(3) For the EEG-fNIRS causal features, the accuracies between EEG signals in θ, α, β, and γ frequency bands and fNIRS signals are 45.90%, 45.81%, 46.40%, and 67.29%, respectively. It is worth noting that the recognition accuracies of the γ frequency band EEG signal are significantly higher than those of the other frequency bands. This observation suggests a stronger cross-modal coupling in the γ frequency band EEG signal and supporting the presence of an informative causal relationship between EEG and fNIRS. These observations motivate leveraging causal features in the subsequent multimodal fusion.

### 5.3. Emotion Recognition Performance of Combination Features

Table 2 summarizes recognition accuracies for different combinations of EEG, fNIRS, and EEG-fNIRS features using the proposed 1D2D-CNN model.

The results demonstrate that each combined feature outperforms its corresponding single-modality baseline. Specifically, combining EEG with HbO_2_, HbR, and HbO_2_ + HbR yields accuracies of 94.65%, 93.66%, and 94.76%, i.e., improvements of 4.11%, 3.12%, and 4.22% over the single EEG feature, respectively. Similarly, the EEG + EEG-fNIRS combination achieve improvements of 2.89% and 23.12% over the single EEG and EEG-fNIRS features, respectively, and the HbO_2_ + HbR + EEG-fNIRS combination show increases of 6% and 20.77% in accuracy over the single HbO_2_ + HbR and EEG-fNIRS features, respectively. Specifically, the three-way fusion achieves the highest emotion recognition accuracy of 94.86%, which is 4.32%, 8.78%, and 24.55% higher than the single EEG, HbO_2_ + HbR, and EEG–fNIRS features, respectively. These findings demonstrate strong cross-modal complementarity.

Notably, although the single EEG-fNIRS causal feature yields lower standalone accuracy, it effectively enhances the recognition performance when fused with EEG and fNIRS features. In addition, comparisons across single frequency-modality features indicate heterogeneous contributions to emotion recognition, providing an empirical basis for the proposed MFA-CNN.

### 5.4. Emotion Recognition Performance of the Proposed MFA-CNN Models

To access the contributions of each component in the MFA-CNN model, we conducted ablation studies with the following variants:

(1) 1D2D-CNN: backbone only, without any additional modules;
(2) 1D2D-CNN (MFA): backbone + MFA only; μ=0 in Equation (1);
(3) 1D2D-CNN (WML): backbone + WML only;
(4) MFA-CNN (Unweighted): full model with equal local weights, i.e., $p_{f}\;=\;p_{e}\;=\;p_{ef}\;=\;1$ in Equation (15);
(5) MFA-CNN: proposed full model with learned loss weighting.

Table 3 shows the emotion recognition results and training time of different methods. Compared with 1D2D-CNN, integrating only the MFA or the WML module alone improves the EEG(θ+α+β+γ) + fNIRS recognition accuracy by 0.96% and 0.61%, respectively. This indicates that the proposed MFA module better captures and utilizes the modality–frequency information, and the WML provides beneficial auxiliary supervision. In addition, the full MFA-CNN achieves the highest emotion recognition accuracy of 98.65%, outperforming the 1D2D-CNN, 1D2D-CNN(WML), 1D2D-CNN(MFA), and MFA-CNN (unweighted) by 1.89%, 1.03%, 1.38%, and 0.64%, respectively.

Table 3 also presents the model parameters and training times, while the computational overhead of the proposed variants is negligible. Training times are nearly identical across models—624 s for 1D2D-CNN and 632.6 s for MFA-CNN—indicating that performance gains are achieved without materially increasing runtime. Parameter counts show the same pattern: 1D2D-CNN and 1D2D-CNN(WML) each contain 128,972,816 parameters, while models incorporating the MFA module add just 72 parameters, a practically imperceptible increase. These results demonstrate that MFA-CNN improves recognition accuracy while maintaining comparable computational complexity and model size.

To quantify the contributions of different feature combinations, we visualized the attention weights learned by MFA-CNN. The average attention weights were 0.96, 0.94, 1.04, 1.01, 0.95, 0.93, 1.03, 0.89, and 0.90. Among them, EEG(α), EEG(β) and EEG(α)-fNIRS features received the largest weights, whereas EEG(β)-fNIRS and EEG(γ)-fNIRS were the lowest. This indicates that the model relies more on low-frequency cross-modal coupling and discriminative EEG information in the α and β bands, consistent with the slow time course of NVC and established emotion-related EEG rhythms.

Moreover, a Friedman test across five schemes was significant ($\chi^{2}\left(4\right)=88.304$, $p<10^{-6}$), with a moderate effect size (Kendall’s W=0.442). Post hoc Wilcoxon signed-rank tests with Holm correction showed that MFA-CNN significantly outperformed all other schemes (all adjusted $p<10^{-6}$); effect sizes (Cliff’s δ) were large to very large (|δ| up to 0.96).

### 5.5. Performance Comparisons for Latest Schemes

In this section, we described the comparisons of the proposed MFA-CNN with several state-of-the-art methods on the TYUT 3.0 emotion dataset. To ensure fairness, all methods used the same data division and fivefold cross-validation. Table 4 presents the performance comparison of recognition accuracy and standard deviation, with the proposed MFA-CNN achieving the highest overall accuracy 96.85%.

(1) Compared with Cascade+SVM, Cascade+CNN, and Cascade+GCN, the MFA-CNN improves accuracy by 10.73%, 4.52%, and 5.68%, respectively. This is mainly because those baselines rely on feature stacking for fusion and do not explicitly model the relative importance of modalities or EEG frequency bands. In contrast, MFA-CNN introduces directed cross-modal information flow via GC features and applies MFA for adaptive reweighting, thereby substantially enhancing emotion recognition performance.

(2) Relative to GNN-fusion and weighted fusion+GCN, the MFA-CNN increases overall accuracy by 2.52% and 1.70%, respectively. Although graph models capture inter-channel and regional relationships, these are typically correlation or coherence measures and underrepresent directed neurovascular coupling. MFA-CNN embeds the GC relationship of EEG-fNIRS signals as a structural prior and performs explicit selection at both modality and frequency levels, which is more consistent with the physiological mechanisms of EEG-fNIRS.

(3) Compared with algebraic fusion models such as tensor fusion and p-order polynomial fusion, MFA-CNN improves accuracy by 3.94% and 3.11%, respectively. While algebraic fusion can capture cross-modal interactions, it is prone to dimensional explosion and noise amplification, making it sensitive to regularization and data scale. MFA-CNN uses a 1D–2D CNN architecture to encode temporal and spatial information and couples this with a WML method to balance supervision across branches, thereby avoiding these drawbacks.

(4) Compared with capsule-network variants such as MLF-CapsNet, ST-CapsNet, and MBA-CF-cCapsNet, MFA-CNN consistently achieves the best accuracies. Capsule networks are effective for hierarchical and pose-invariant representations, but they often lack physiological priors and explicit frequency-level constraints in multimodal settings. MFA-CNN integrates causal priors with modality–frequency attention, focusing discriminative power on emotion-sensitive bands and key modality combinations.

(5) Compared with the Transformer method, MFA-CNN achieves a 10.01% improvement. This is primarily because without explicit modality–frequency priors, Transformers struggle to learn stable cross-modal coupling and are more sensitive to hyperparameters and dataset size.

**Figure 7:**
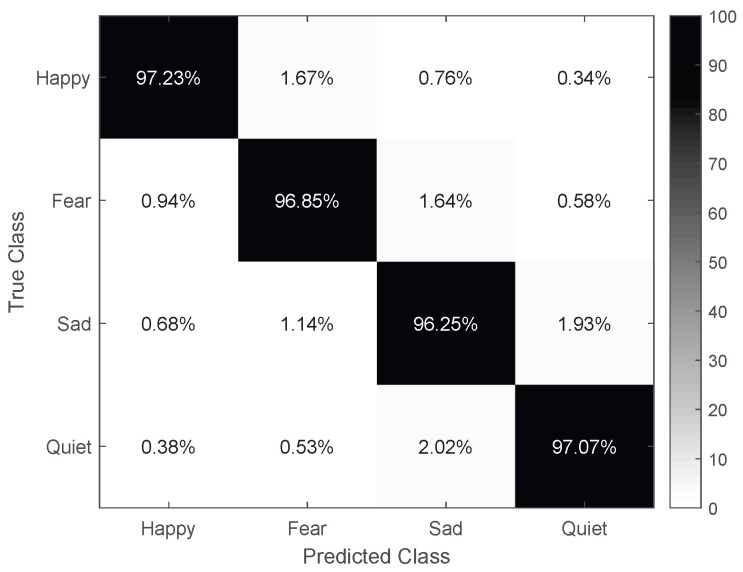
The confusion matrix of the MFA–CNN.

Furthermore, Figure 7 presents the confusion matrix of the MFA-CNN model, showcasing its exceptional classification accuracy for all four emotion types. Although there were a few misclassifications, they were negligible. Notably, the emotions sad and calm (1.64% and 1.14% confusion, respectively) alone with happy and fear (1.67% and 0.94% confusion, respectively) were more susceptible to confusion. This might be attributed to sad and calm both being low-activation emotion types, while happy” and fear are high-activation types.

### 5.6. The Subject–Independent Experiments

The results in the previous subsections demonstrate that MFA-CNN achieves strong emotion recognition performance under the subject-dependent setting. To further assess its generalization and provide a comprehensive view of performance across different application scenarios, we conducted subject-independent experiments on the TYUT-3.0 dataset using a leave-one-subject-out (LOSO) protocol. In each fold, data from 49 participants were used for training, and data from the held-out participant were used exclusively for testing.

Table 5 shows the recognition results of different fusion methods, and the MFA-CNN achieves higher accuracy than does representative fusion baselines from the literature. As expected, due to the pronounced distribution shift between training and testing subjects in the LOSO setting, absolute accuracies are lower than are those in the subject-dependent experiments. Nevertheless, MFA-CNN remains the top performer.

## 6. Conclusions

In this paper, we present MFA-CNN, a multimodal EEG-fNIRS emotion-recognition framework that couples a 1D–2D CNN backbone with an MFA module and WML strategy. First, we quantified the causal coupling between EEG and fNIRS via the GC method and verified that incorporating GC-derived cross-modal features enhances recognition performance. The network fuses EEG, fNIRS, and EEG-fNIRS causal features, while MFA adaptively assigns weights across modalities and frequency bands, and WML jointly optimizes global and branch-specific losses. Experiments on the TYUT-3.0 dataset show that MFA-CNN consistently outperforms representative EEG-fNIRS fusion methods in both accuracy and stability. Beyond performance, the GC analysis provides a quantitative perspective on neurovascular coupling during affective processing, offering evidence for the underlying neural mechanisms and indicating a promising applicability to diverse EEG-fNIRS-based brain–computer interface tasks. Practically, our findings can be leveraged for driver fatigue monitoring, workplace stress assessment, student mental-workload monitoring, and clinical rehabilitation scenarios such as emotion and pain evaluation.

Future work will pursue cross-subject emotion recognition by incorporating advanced transfer learning and domain adaptation techniques (e.g., subject-invariant representations and zero-shot adaptation). In parallel, guided by the MFA-identified modality–frequency combinations most sensitive to emotion, we will explore lightweighting of the emotion recognition model, deepen understanding of the brain’s emotion-processing mechanisms, and develop an efficient, low-latency online EEG–fNIRS BCI system.

## Figures and Tables

**Table 1:** Parameters of the MFA-CNN model.

Layers	Filter Size			Stride	Padding
	**GC(EEG)**	**HbO_2_ + HbR**	**GC(EEG-fNIRS)**		
Conv1	32@3×3	32@3×1	32@3×3	1	1
ReLU					
Conv2	64@3×3	64@3×1	64@3×3	1	1
ReLU					
Conv3	128@3×3	128@3×1	128@3×3	1	1
ReLU					
FC1	128×4	128	128×4		
Concatenate	128×9				
FC2	4				
ReLU					
Softmax	4				

**Table 2:** Emotion recognition accuracy of different combination features (%).

Features	EEG(θ)	EEG(α)	EEG(β)	EEG(γ)	EEG(θ+α+β+γ)
EEG + HbO_2_	84.56/3.60	85.07/3.19	84.16/3.98	89.01/9.12	94.65/4.56
EEG + HbR	84.87/3.53	84.05/3.95	83.90/4.54	82.68/7.30	93.66/4.92
EEG + (HbO_2_ + HbR)	86.64/3.21	87.61/3.85	86.51/4.21	90.52/4.12	94.76/3.81
EEG + (EEG-fNIRS)	88.86/5.37	89.02/5.37	88.93/5.45	88.86/5.05	93.43/4.88
(HbO_2_ + HbR) + (EEG-fNIRS)	87.05/7.02	89.95/3.90	89.55/5.52	85.56/8.25	92.08/6.65
EEG + (HbO_2_ + HbR) + (EEG-fNIRS)	86.71/5.13	88.95/4.97	86.54/4.85	89.15/5.92	94.86/3.81

**Table 3:** Ablation experimental results of the MFA-CNN model (%).

Method	EEG Band + fNIRS				EEG(θ+α+β+γ) + fNIRS		
	**EEG(θ) + fNIRS**	**EEG(α) + fNIRS**	**EEG(β) + fNIRS**	**EEG(γ) + fNIRS**	**Accuracy/SD**	**Time/s**	**Parameters**
1D2D-CNN	86.71/5.13	88.95/4.97	86.54/4.85	89.15/5.92	94.86/3.81	624	128,972,816
1D2D-CNN (MFA)	90.07/4.42	88.57/4.74	90.67/2.57	89.72/3.31	95.82/3.45	626.5	128,972,888
1D2D-CNN (WML)	89.52/3.84	88.46/4.01	90.52/2.99	90.12/3.56	95.47/3.55	624.5	128,972,816
MFA-CNN (Unweighted)	91.97/4.37	90.60/4.74	91.70/2.57	90.75/3.32	96.21/3.32	628.6	128,972,888
MFA-CNN	92.07/4.28	90.28/4.60	92.09/2.40	92.46/2.01	96.85/1.92	632.6	128,972,888

**Table 4:** Comparison of performance between different emotion recognition models (%). Entries in the last column are mean/SD.

Feature Fusion/Model	Happy	Fear	Sad	Calm	Acc./Std
Cascade+SVM	90.74	82.01	83.74	87.90	86.12/6.71
Cascade+CNN	93.46	94.21	89.13	92.51	92.33/4.94
Cascade+GCN	92.52	90.19	90.71	91.06	91.17/4.01
Weighted fusion+GCN [44]	98.70	91.94	91.32	98.55	95.15/3.15
GNN-fusion [44]	94.11	94.85	93.55	94.81	94.33/2.98
Tensor fusion [45]	92.41	91.60	91.83	95.79	92.91/3.00
P-order polynomial fusion [45]	94.62	94.92	92.97	92.44	93.74/3.14
SG_SC [46]	97.10	96.60	95.40	97.50	91.70/3.02
TC-Net [47]	85.62	89.35	83.84	88.54	86.84/9.25
MLF-CapsNet [48]	94.48	94.79	93.46	95.86	94.65/3.80
ST-CapsNet [49]	93.57	95.02	93.04	94.42	94.01/2.95
Transformers [50]	85.62	89.35	83.84	88.54	86.84/9.25
MBA-CF-cCapsNet [29]	96.57	97.31	95.02	97.76	96.67/2.68
MFA-CNN (ours)	97.05	96.66	96.07	96.88	96.85/1.92

**Table 5:** The subject–independent results of different fusion methods (%).

Method	Acc.	Std
Cascade+SVM	47.85	14.51
Cascade+CNN	53.67	11.35
Cascade+GCN	55.85	10.46
*p*-order polynomial fusion [45]	52.33	10.80
Tensor fusion [45]	50.46	10.12
Weighted fusion + GCN [44]	52.80	11.50
GNN-fusion [44]	53.98	12.95
Transformers [50]	53.87	10.81
MFA-CNN (ours)	58.59	9.89

## Data Availability

The data presented in this study are available on request due to privacy and ethical reasons. To obtain access, please download and complete the license agreement at https://gitee.com/tycgj/enter (accessed on 1 September 2025). After completing the form, kindly email it to 2023025@tyust.edu.cn. A download link will be provided via email after your application is reviewed.

## Abbreviations

The following abbreviations are used in this manuscript:

EEG	electroencephalography
fNIRS	functional near-infrared spectroscopy
GC	Granger causality
CNN	convolutional neural network
MFA	modality–frequency attention
HbO_2_	oxyhemoglobin
HbR	deoxyhemoglobin
GCN	graph convolutional network
WML	weighted multi-loss
SVM	support vector machine
